# Fast, Slow, or Not at All: The Extracellular Matrix Controls Movement of Signaling Molecules

**DOI:** 10.1371/journal.pbio.1001362

**Published:** 2012-07-17

**Authors:** Richard Robinson

**Affiliations:** Freelance Science Writer, Sherborn, Massachusetts, United States of America

**Figure:**
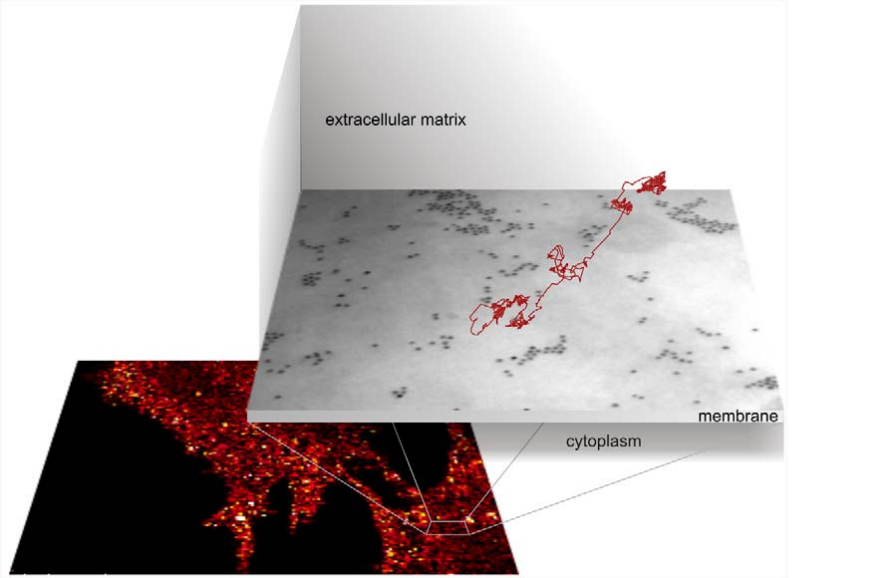
Montage of a false color photothermal image of FGF2-nanoparticles on two fibroblast cells and of an electron micrograph showing the clustering of FGF2-nanoparticles (black dots) with a superimposed track of an FGF2 (red line).

From elementary school biology on up, almost every diagram of every cell includes a sharp line dividing inside—a hive of well-ordered biochemical activity—from outside, usually depicted as a void, with perhaps the occasional signaling molecule drifting randomly and alone toward the plasma membrane. But it just isn't so. The extracellular matrix is extensive, complex, and vital to both the structure and function of the cell. This gelatinous network of sugars and proteins not only links the cell to its neighbors, it regulates incoming molecular traffic by binding and transporting a wide variety of proteins, helping to control the movement of hormones, cytokines, and growth factors, among others, and establishing gradients of these compounds that are important in the development of the organism and for fighting infections.

Proteoglycans are key ingredients of the matrix, and are the dominant molecule closest to the plasma membrane. Each is composed of a “core protein”, some of which are embedded in the membrane, and each is linked to many sugar chains that extend outward. One of the most common and important chains is heparan sulfate (HS), whose repeating sugars and dangling sulfates create multiple binding sites for many different extracellular proteins. Multiple proteins may bind to the same spot on HS with varying affinities, and a single protein may bind at several different sites. The richness of this arrangement has made studying the trafficking of proteins within the matrix a challenge, and little is known about the details of that movement. In this issue of *PLoS Biology*, Laurence Duchesne, David Fernig, and colleagues use two imaging techniques to locate and track the movement of a growth factor within the matrix, and demonstrate that the spatial organization of binding sites on HS governs both the rate and distance traveled by the factor.

The authors first attached gold nanoparticles to fibroblast growth factor 2 (FGF2), which binds to HS, and showed that the gold, which allowed FGF2 to be imaged, did not interfere with the transport or function of the protein. Using transmission electron microscopy, they found that FGF2 bound to HS in clusters, rather than being evenly or randomly spread throughout the matrix, and that more than 99% of the protein was bound to HS, rather than to the FGF receptor, the signaling molecule that is embedded in the plasma membrane.

Next they tracked the movements of the nanoparticles by using photothermal heterodyne imaging, a technique in which energy released from a stimulated particle is detected, allowing its movements to be mapped over intervals of tens of minutes or even longer. Previous work by these authors and others has shown that once within the matrix, very little FGF2 departs again for the bulk culture medium, but it does move within the matrix. Here, they found that FGF2 movements could be classified as one of 5 types: immobile or highly confined, confined, simple diffusion, slow directed diffusion, and fast directed diffusion. Most molecules spent most of their time (approximately 83%) either immobile or confined. Simple diffusion occupied 13% of their time, while 3% of the time they were involved in slow directed diffusion. Fast directed diffusion was quite rare.

To understand better the nature of the confined state, the authors compared the motion of FGF2 in the matrix of living cells to that of fixed cells, in which the membrane-bound proteins are immobile but the HS chains tethered to them are still free to move about. Not surprisingly, the diameter of confinement (i.e., the distance over which the FGF2 could move) was greater in live cells than in fixed cells (106 nm versus 94 nm). But increasing the concentration of FGF2 by 10-fold produced an interesting effect: it shrank the diameter of confinement for fixed cells, but increased it for live cells. The authors suggest that for fixed cells, the increased concentration meant that most local FGF2-binding sites would be occupied, reducing the ability of individual FGF2 molecules to move about. For live cells, however, the increased concentration of FGF2 may displace more non-FGF2 proteins from their binding sites (not as likely in fixed cells, due to the protein-stabilizing effects of the fixation process), allowing any one FGF2 molecule to move more readily among binding sites. Other effects may also contribute, they note.

Slow directed diffusion occurred in both living and fixed cells, but was twice as common in the former. The authors suggest that the major mechanism involved is disengagement of a single FGF2 from one binding site and reengagement with a neighboring site on HS molecules, which may be spatially organized to form a path for directed movements. The overall movement of any one FGF2 would then be a combination of random movements among local networks of binding sites (confined movement), and movement along paths (directed diffusion).

The authors note that such a system of confinement and path-taking may not be restricted to FGF2, but is likely to characterize the movement of many molecules in the extracellular matrix, and is likely to be important for controlling communication between the environment and individual cells.

**Duchesne L, Octeau V, Bearon RN, Beckett A, Prior IA, et al. (2012) Transport of Fibroblast Growth Factor 2 in the Pericellular Matrix Is Controlled by the Spatial Distribution of Its Binding Sites in Heparan Sulfate. doi:10.1371/journal.pbio.1001361**

